# Depression in Hepatitis B and C, and Its Correlation With Hepatitis Drugs Consumption (Interfron/Lamivodin/Ribaverin)

**Published:** 2013

**Authors:** Shahriar Alian, Abbas Masoudzadeh, Talayeh Khoddad, Amir Dadashian, Reza Ali Mohammadpour

**Affiliations:** 1 Department of infectious disease, Mazandaran university of Medical Science.; 2 Psychiatry and Behavioral Sciences Research Center, Department of Psychiatriy, Mazandaran University of Medical Science, Sari, Iran.; 3 Clinical Research Development Unit of Imam Khomeini hospital, Mazandaran University of Medical Science.; 4 Medical doctor, Mazandaran University of Medical Science.; 5 Department of Biostatistics, Mazandaran University of Medical Science.

**Keywords:** Chronic Hepatitis B, Chronic Hepatitis C, Depression Prevalence

## Abstract

**Objective**: Chronic infection of hepatitis B and hepatitis C are considered as the most important infectious diseases, which lead to drastic consequences such as liver dysfunction. Depression is a psychiatric disorder which is concomitantly present in these patients, and decreases the patients’ quality of life. It may lead to suicide, homicide or intentional transmission of infectious to others. Medical treatment with interferon can also lead to depression which is comparable to the depression caused by disease.

**Methods: **We performed a cross sectional study on 205 patients with hepatitis B and hepatitis C infection. We aimed to determine the prevalence of depression via Beck Depression Inventory (BDI), and its correlates with hepatitis drugs.

**Results:** Of 205 patients, 154 cases had hepatitis B and 51 cases had hepatitis C infection. The frequency of depression was 68% in hepatitis B and 86% in hepatitis C infected patients (p<0.05). The frequency of mild depression was 14%, moderate depression was 57.3% and severe depression was 28.7% (p<0.05). Depression frequency in Interferon recipients was 100%, in interferon-ribavirin recipients was 94.4%, in lamivudine recipients was 64%, and in patients that receive no drug was 66.7%. Depression prevalence was significantly higher among those on interferon therapy (p<0.05).

**Conclusion: **There is a high prevalence of depression among patients with hepatitis B and hepatitis C infection, especially patients on interferon therapy. Hence these patients should be repeatedly evaluated for depression.

**Declaration of Interest:** None.

## Introduction

Hepatitis B is one of the most common diseases in Iran. Nearly 3% of the populations are carrier and approximately 200-300 thousands of people have chronic hepatitis B disease. Also hepatitis C has infected 0.05% of Iranian people (1).

Depression is also a prevalent disorder which can decrease the quality of life and social activities in these patients. Intentional transmission of hepatitis may also occur by high risk behaviors such as unprotected sexual activities (2-4).

Depression and anxiety are prevalent in patients with chronic hepatitis B and chronic hepatitis C. It is mainly due to impaired health related quality of life (especially social activities) and fear of disease complications (5). So it has a great impact on the society as there is a high prevalence of hepatitis in Iran. As a consequence the physical cooperation and therapeutic compliance of patients for medical treatment and follow up will decrease. These conditions may lead them to a vicious cycle (6,7). It is also shown that the patients’ fatigue is related to depression (8).

In addition, depression and anxiety are major complications of interferon therapy (main agent in hepatitis management) (3,9-11). Furthermore, some studies indicated that depression is one of the serious side effects of new anti-viral therapy which can be used for chronic hepatitis C treatment. This could also be another reason of a higher prevalence of depression in chronic hepatitis C compared to chronic hepatitis B (12,13).

It is noteworthy to say that even among of patients who received no medical treatment, patients with chronic hepatitis C have shown depressive symptoms more than chronic hepatitis B (14,15).

To date we are unaware of any study in evaluating depression as a complication of new anti-viral therapy in chronic hepatitis B and C. In addition, there isn’t any protocol to refer patients with chronic hepatitis B and C for psychological assessment Here we aimed to study depression and its association with interferon and oral anti-viral drugs in patients with chronic hepatitis B and C infection. 

## Materials and Methods

We performed a cross sectional study on patients with chronic hepatitis B and C infection attending the infectious clinic of Raze hospital in Ghaemshahr, and gastrointestinal clinic of Imam Khomeini hospital in Sari-Iran. Demographic and anthropometric properties of participants were recruited using a questionnaire. These data was completed by general physician with history, physical examination and previous laboratory documents.

The depression was evaluated using the Farsi version of Beck Depression Inventory (BDI). BDI, created by Dr. Aaron T. Beck, is a 21-question multiple-choice self-report inventory one of the most widely used instruments for measuring the severity of depression. Its development marked a shift among health care professionals, who had until then viewed depression from a psychodynamic perspective, instead of it being rooted in the patient's own thoughts (16). This questionnaire has been examined by several studies in the past years. The first validity assessment of Persian translation of BDI in Iran has been done by Wahabzadeh 1973 and its validity reported high (70%). In addition, other assessment in past years by Tashakkori, Mehryar 1994, Chegini 2002 reported high validity ofBDI (70-90%). In its current version the inventory is designed for individuals aged 13 and over, and is composed of items relating to symptoms of depression such as hopelessness and irritability, cognitions such as guilt or feelings of being punished,as well as physical symptoms such as fatigue, weight loss, and lack of interest in sex (17).

The BDI was completed by patient. In this test, one response should give to each question. Any response receives 0-3 score. Total score will be 0-63.

In our study, severity scores of depressive disorder were assessed as: 0-9, minimal; 10-18, mild; 19-29, moderate; and 30-63, severe (16).

The study design was explained for all the participants and they signed written informed consent before participation. The study was conducted according to the principles of the declaration of Helsinki. Each participant responded to questions individually. The answers were then evaluated by a psychologist the score of patients' depression were determined.

Exclusion criteria was the presence of any psychiatric disorder on admission patients with Score higher than 29, were advised to consult a psychiatrist.

SPSS software version 16 was used for analysis. Central and dispersion parameters were used to description of the population. Independent sample t-test was employed to compare the quantitative data between 2 groups.

One way ANOVA was used to compare the quantitative data between the groups and CHI-SQUARE to comparing Qualitative data in 2 groups.

Pearson correlation used for determination of quantitative data’s correlation and p<0.05 was considered statistically significant.

## Results

Two hundred twenty seven patients were evaluated, 14 patients were excluded from study because of positive history of psychiatric disorders and drug usage. Two hundred thirteen patients entered and received 

the standard Beck questionnaire, 205 of them filled the questionnaire completely, 161 of them were (78.5%) were men and 44 (21.5%) of them were women.

The mean age of the patients was 38.27±9.52 years old with maximum 63 yr. and minimum 18 yr.

The mean age of patients in group with no depression was 48.76±8.2 yr., in group with mild depression was 39.48±8.21 yr., in group with moderate depression was 35.37±5.5 yr. and in group with severe depression was 30.07±6.06 yr.; (p<0.05).

150 patients (73.2%) had different degrees of depression117 men (72.7% of men) and 33 women (75% of women) were depressed, which was not statistically significant. (Table 1)Moderate depression was the most common type of depression in 2 gender groups (men and women) and also in groups of chronic hepatitis Band C. The mean duration of disease diagnosis was38.5±17.92 months with maximum 10yr and minimum 12 months.

The mean duration of diagnosis in patients with no depression was 52.44±21.01 months, in patients with mild depression were 46.67±13.31 months, in patients with moderate depression was 36.76±10.76 months and in patients with severe depression was 24.16±12.16 months. There was significant difference between 2 groups of patients (patients with mild and no depression) with others (patients with moderate and severe depression) (p<0.05).

154 cases (75.1%) had chronic hepatitis B and 51 cases (24.9%) had chronic hepatitis C. (Table 2 , 3).

**Table.1:** Different degrees of depression frequency in two gender groups

**Summation**	**Severe depression** **(Beck score30-63)**	**Moderate depression** **(Beck score19-29)**	**Mild depression** **(Beck score10-18)**	**No depression** **(Beck score0-9)**	**Sex**
44(100%)	10(22.7%)	15(34.1%)	8(18.2%)	11(25%)	**Female**
161(100%)	33(22.5%)	71(44.1%)	13(8.1%)	44(27.3%)	**Male**
205(100%)	43(21%)	86(42%)	21(10.2%)	55(26.8%)	**Summation**

p> 0.05

**Table.2:** Distribution of depression based on type of disease and consumptive drugs

**Drug groups**	**Type of chronic hepatitis**	**Depression existence**		**Summation**
		**positive**	**Negative**	
**No drug**	B C Summation	3(33.3%) 5(33.3%) 8(33.3%)	6(66.7%) 10(66.7%) 16(66.7%)	9 15 24(11.7%)
**Interferon**	B	0	20(100%)	20(9%)
**lamivudine**	B	45(36%)	80(64%)	125(60.9%)
**interferon+ribaverin**	C	2(5.6%)	34(94.4%)	36(17.5%)

p< 0.05

**Table3:** depression frequency in patients with chronic hepatitis B and C

**Summation**	**Depression existence**		**Type of chronic hepatitis**
	**positive**	**Negative**	
154(100%)	106(68.8%)	48(31.2%)	**B**
51(100%)	44(86.3%)	7(13.7%)	**C**
205(100%)	150(73.2%)	55(26.8%)	**Summation**

p=0.015

X^2^ =5.938

Fisher's exact test = 0.010

The mean score of BDI was 22.27±11.88. Maximum score was 56 and minimum score was 2.

The mean score of beck test in men was 22.83±11.78 and in women was 22.05±12.36. This was statistically significant (p>0.05).

The mean score of BDI in patients with chronic hepatitis B was statistically lower than patients with chronic hepatitis C (21.31±11.7 vs. 26.76±11.52; (p<0.05).

The mean score of beck test in patients with no drug consumption was 21.3±12.50; in interferon recipients was 21.3±12.50; in lamivudine recipients was 20.30±12.10 and in interferon+ lamivudine recipients was 29.06±10.37. There was significant difference in mean score of BDI between interferon recipients and patients with no drug consumption (p<0.05).

There was meaningful difference statistically in the mean score of BDI between interferon recipients and lamivudine recipients (p<0.05).

The mean score of BDI in interferon recipients and interferon+ribavrin recipients does not have significant difference (p>0.05).

## Discussion

Here we showed that there is statistically significant difference between patients with chronic hepatitis B and patients with chronic hepatitis C in different degrees of depression.

Mauro G Carta and et al.(14) evaluated 135 patients with chronic hepatitis C and 76 patients with chronic hepatitis B for and the presence of major depressive disorder. They demonstrated a higher prevalence of major depressive disorder in chronic hepatitis C compared to chronic hepatitis B and control group. Eventually, correlation between chronic hepatitis C and major depressive disorder (based on international diagnostic criteria), was independent from the interferon therapy. This may be due to less therapeutic options, more social fear and non-significant prognosis of chronic hepatitis C that lead to more depression rate (14, 15).

In our study 72.7% of men and 75% of women with chronic hepatitis B or hepatitis C had different degrees of depression, and there was no significant difference between them. While Loftis and et al. reported depression prevalence is 15% in general population, which is significantly higher in women (5% in men and 25% in women) (6). The highest prevalence of depression in general population was reported in Uganda by WHOM (21% in men and 17% in women). In Iran Attari A. and et al. reported 32% depression prevalence in general population of Sahreza (32% in men and 34% in women) (18). According to these results, the prevalence of depression in chronic hepatitis B or C was more than general population, while there was no significant difference between men and women. It may be due to the same effect of disease and/or drug side effects in men and women.

In our study, the mean age of patients with no depression was 48.76 yr., and the mean age of patients with depression was 43.32 yr., and the mean age patients with severe depression were significantly lower than patients with mild depression or no depression. There were significant differences between non-depressed patients and depressed in the mean duration of disease diagnosis. So the mean duration of patients' awareness of disease in patients with mild and no depression was significantly more than patients with moderate and severe depression. It also has a direct effect on the BDI score. Kraus MR and et al (15) who evaluated the emotional state (depression, anxiety) in 113 chronic hepatitis C patients without progressed liver disease, showed older patients (≥50 years) were more significantly more depressed (P=0.024) and patients whom their diseases was recently diagnosed (>4 weeks, <6 months) had significantly a lower depression scores (p=0.003).

Our findings may be because of making adaptation to disease proceeding and treatments, despite of more complications that may occur in older patients. So an older age and a longer diagnosis can decrease the degrees of depression. Younger patients are more severely influenced by the awareness of their diseases. Even they may be stigmatized and banished from society. So they may be deprived from usual activities. Because of these reasons, they suffer from depression  symptoms more than older patients.

In our study, 66.7% of patients with no drug consumption, 100% of patients with interferon consumption, 94.4% of patients with interferon+ribavirin consumption and 64% of patients with lamivudine consumption were depressed. There was meaningful difference between no drug or lamivudine recipients and interfrone recipients. These results may be because of interferon effects. 

In Piero Amodio and et al. study in 2005 (9), twenty patients with chronic hepatitis C were evaluated while they were under treatment by α-IFN. Patients were assessed about depression by beck test questionnaire at baseline and 2 and 6 months after the beginning of treatment. BDI scores of patients were increased by treatment 6 months after beginning of treatment. At the end, they concluded that α-IFN treatment makes mild symptoms of depression

In addition, chronic stress may play role in depression development in chronic hepatitis B and C. It can be due to an increase in the plasma cortisol levels (as a stress associated agent) and impaired serotonin uptake (as a known agent that plays role in depression).Tafet GE et al. (19) demonstrated chronic stress plays a critical role in the beginning and development of depression. They demonstrated Plasma cortisol level in normal persons is peaked in morning and decreases throughout the day, but plasma cortisol level in patients with major depression disorder is peaked earlier and has no decline in its level throughout the day. They also observed a substantial increase in the afternoon cortisol level in the blood of patients with major depression disorder. This elevation has been described as a characteristic of the hypercortisolism that is observed in chronic stress. Their study showed a correlation between hypercortisolemia and impaired serotonergic neuronal transfer in central nervous system and considered this correlation as its mechanism.

According to our study, patients with chronic hepatitis B and C need more psychological assessments for the diagnosis of depression or other psychiatric disorders. Early diagnosis and treatment of depression in chronic hepatitis B and C can prevent the decrease in the quality of life and patients’ familial and social relationship and it can prevent high risk behaviors in these patients.

In patients who receive interferon, it should also be considered that depression may occur as interferon side effect. Also patients with chronic hepatitis C that have history of interferon-induced depression can be treated with peginterferon / ribavirin and anti-depressant drugs for prophylaxis of depression (12).So, educational programs should be performed for patients with chronic hepatitis B and C to increase their quality of life and social activities.

According to lack of control healthy group in our study, we suggest to do wider studies with control group.

## Authors’ Contributions

ShA conceived and designed the evaluation. AM participated in designing the evaluation. TKh collected the clinical data, interpreted them, drafted and revised the manuscript. AD re-evaluated the clinical data and revised the manuscript. RAM did the statistical analyses. All authors read and approved the final manuscript.
